# Workflow Modelling and Analysis Based on the Construction of Task Models

**DOI:** 10.1155/2015/481767

**Published:** 2015-01-29

**Authors:** Glória Cravo

**Affiliations:** ^1^Center for Linear Structures and Combinatorics, University of Lisbon, 1649-003 Lisbon, Portugal; ^2^Center of Exact Sciences and Engineering, University of Madeira, Funchal, 9020-105 Madeira, Portugal

## Abstract

We describe the structure of a workflow as a graph whose vertices represent tasks and the arcs are associated to workflow transitions in this paper. To each task an input/output logic operator is associated. Furthermore, we associate a Boolean term to each transition present in the workflow. We still identify the structure of workflows and describe their dynamism through the construction of new task models. This construction is very simple and intuitive since it is based on the analysis of all tasks present on the workflow that allows us to describe the dynamism of the workflow very easily. So, our approach has the advantage of being very intuitive, which is an important highlight of our work. We also introduce the concept of logical termination of workflows and provide conditions under which this property is valid. Finally, we provide a counter-example which shows that a conjecture presented in a previous article is false.

## 1. Introduction

A workflow is an abstraction of a business process that consists on the execution of a set of tasks to complete a process (e.g., hiring process, loan application, and sales order processing). Tasks represent unities of work to be executed that can be processed by a combination of resources, such as a computer program, an external system, or human activity.

Recall that a business process is a collection of interconnected tasks that takes one or more kinds of input and creates an output that is of value to the customers. The construction of process models is very often a difficult accomplishment for humans since their design can be logically incorrect and enclose errors. So the development of tools to support the design of business processes is indispensable and needs to be based on a solid theory. We believe that our technique is appropriate to model workflows.

Workflows have been successfully deployed to various domains, such as bioinformatics, healthcare, the telecommunication industry, the military, insurance, school administration, mobile computing, systems management, multidata bases, Internet, application development, object technology, operating systems, and transaction management.

In the present paper, we use Graph Theory and Propositional Logic to describe the structure of workflows. It is important to point out that a workflow describes all of the tasks needed to achieve each step in a business process. Documents, information, work orders, reports, and so forth are passed from one task to another for action, according to a set of rules defined by the workflow. Employees or automated applications are the entities that carry out the execution of tasks.

In particular we model workflows as trilogic acyclic directed graphs. The use of trilogic graphs to represent workflows was selected since most business process languages support three types of connectors: AND, OR, and XOR. It is important to emphasize that the inclusion of workflows with OR vertices is a significant advantage of our approach.

Furthermore, our approach is based on the execution of all tasks present on the workflow. This analysis has the advantage of being simple and very intuitive. On the other hand, we can create new models based on the existing ones.

Besides our formal framework allows checking the logical termination of workflows. The logical termination is an important property for workflows because it is indispensable to know if a workflow, such as the hiring process, will eventually finish. Analyzing the termination of workflows is an important assignment since research and commercial products, such as METEOR and TIBCO, have no support for verification. Errors made at design-time are not detected and result in very costly failures at run-time.

The use of Propositional Logic has the advantage of transforming a workflow into a set of Event-Action (EA) models. Specialized EA models can be easily created to represent new advanced workflow patterns. Afterwards, Propositional Logic and Inference can be carried out on the EA models to analyze properties of workflow models.

It is important to point out that, in the last decade, the rapid increase of business process modelling and management through the adoption of Workflow Management Systems has originated the need for frameworks that can be used to provide a formal technique for defining and analyzing workflows [1–3]. Important advancements have been accomplished in the development of theoretical foundations to allow workflow modeling, verification, and analysis. Several formal methods have been proposed, such as State and Activity Charts [4], Event-Condition-Action rules [5, 6], Petri Nets [7–11], Temporal Logic [12], Markov chains [13], Process and Event Algebras [14, 15], and Six Sigma Techniques [16, 17]. Nevertheless more research is required and specially focused on the use of Graph Theory. Based on this need, we develop our formalism that uses a natural combination of Graph Theory and Propositional Logic to model workflows. Besides, our formalism provides a formal framework based on trilogic acyclic directed graphs that facilitate modeling and analyzing workflows. Finally, our formal framework allows checking the logical termination of workflows.

An important highlight of this paper is the emphasis on the tasks present in the workflow, which allows us to identify easily the dynamism present in the workflow. Finally, we describe the logical termination in a very intuitive form and we present conditions under which this property is valid.

We still provide a counter-example which shows that a conjecture presented in a previous article is false.

## 2. Workflow Modelling and Analysis

This section is devoted to the presentation of our main results. In particular, we start this section by providing the formal definition of a workflow. In other words, we furnish the formal structure of a business process. Notice that this workflow structure can be also found in [18–21]. It is also important to point out that this type of graphs has an input/output logic operator associated with each vertex. Further, we analyze each model present on the workflow and give special emphasis to the execution of all tasks present in a workflow. Besides, we will create new models based on the existing ones. Finally, we will describe conditions under which a workflow logical terminates. In conclusion, our approach allows us to provide a complete description of workflows.

**Definition 1 (see [18–21]).**

A workflow is a trilogic acrylic directed graph WG = (*T*, *A*, *A*′, *M*), where *T* = {*t*_1_, *t*_2_,…, *t*_*n*_} is a finite nonempty set of vertices representing workflow tasks. Each task *t*_*i*_ (i.e., a vertex) has attributed an input logic operator (represented by ≻*t*_*i*_) and an output logic operator (represented by *t*_*i*_≺). An input/output logic operator can be the logical AND (•), the OR (⊗), or the XOR—exclusive-or—(⊕). The set *A* = {*a*_⊔_, *a*_⊓_, *a*_1_, *a*_2_,…, *a*_*m*_} is a finite nonempty set of arcs representing workflow transitions. The transition *a*_⊔_ is the tuple (⊔, *t*_1_) and transition *a*_⊓_ is the tuple (*t*_*n*_, ⊓), where the symbols ⊔ and ⊓ represent abstract tasks which indicate the entry and ending point of the workflow, respectively. Every transition *a*_*i*_, *i* ∈ {1,…, *n*} corresponds to a tuple of the form (*t*_*k*_, *t*_*l*_), where *t*_*k*_, *t*_*l*_ ∈ *T*.

We use the symbol  ′ to reference the label of a transition; that is, *a*_*i*_′ references transition *a*_*i*_,  *a*_*i*_ ∈ *A*. The elements *a*_*i*_′ are called Boolean terms and form the set *A*′.

Given *t*_*i*_ ∈ *T*, the incoming transitions for task *t*_*i*_ are the tuples of the form (*t*_*l*_, *t*_*i*_),  *t*_*l*_ ∈ *T*, and the outgoing transitions are the tuples of the form (*t*_*i*_, *t*_*l*_),  *t*_*l*_ ∈ *T*.

The incoming/outgoing condition of task *t*_*i*_ is the Boolean expression *a*_*k*_1__′*φ* ⋯ *φa*_*k*_*l*__′, where *φ* ∈ {•, ⊗, ⊕},  *a*_*k*_1__′,…, *a*_*k*_*l*__′ ∈ *A*′ and *a*_*k*_1__,…, *a*_*k*_*l*__ are the incoming/outgoing transitions of task *t*_*i*_. The terms *a*_*k*_1__′,…, *a*_*k*_*l*__′ are connected with the logic operator ≻*t*_*i*_,  *t*_*i*_≺, respectively. If task *t*_*i*_ has only one incoming/outgoing transition we assume that the condition does not have logic operator.

An Event-Action (EA) model for task *t*_*i*_ is an implication of the form *t*_*i*_ : *f*_*E*_⇝*f*_*C*_, where *f*_*E*_ and *f*_*C*_ are the incoming and outgoing conditions of task *t*_*i*_, respectively. An EA model has the behavior with two distinct modes: when *f*_*E*_ is evaluated to true, *f*_*C*_ is also evaluated to true; when *f*_*E*_ is evaluated to false, *f*_*C*_ is always false. And the EA model *t*_*i*_ : *f*_*E*_⇝*f*_*C*_ is true if both *f*_*E*_, *f*_*C*_ are true; otherwise it is false. We say that the EA model *t*_*i*_ : *f*_*E*_⇝*f*_*C*_ is positive if its Boolean value is true; otherwise it is said to be negative.

We denote by *M* the set of all EA models present in WG.

Task *t*_*i*_ is said to be executed if the EA model *t*_*i*_ : *f*_*E*_⇝*f*_*C*_ is positive. In this case, task *t*_*i*_ has attributed the Boolean value true.

**Remark 2 .**

Given an expression whose Boolean value is true (resp., false), we simply can represent this fact by 1, (resp., 0).

**Remark 3 .**

Given an EA model *t*_*i*_ : *f*_*E*_⇝*f*_*C*_, if *f*_*E*_ is false, then task *t*_*i*_ disables all its outgoing transitions. Consequently *f*_*C*_ is also false.

Notice that the workflow starts its execution by enabling transition *a*_⊔_, that is, by asserting *a*_⊔_′ to be true. In other words, the workflow starts its execution by executing task *t*_1_.

Notice that *a*_*i*_′ is true if transition *a*_*i*_ is enabled; otherwise *a*_*i*_ is false. Transitions can be enabled by a user or by an external event. If the EA model *t*_*i*_ : *f*_*E*_⇝*f*_*C*_ is negative, then both *f*_*E*_,  *f*_*C*_ are false. In this case, all the transitions of *f*_*C*_ are disabled.

**Example 4 .**

**Figure 1:**
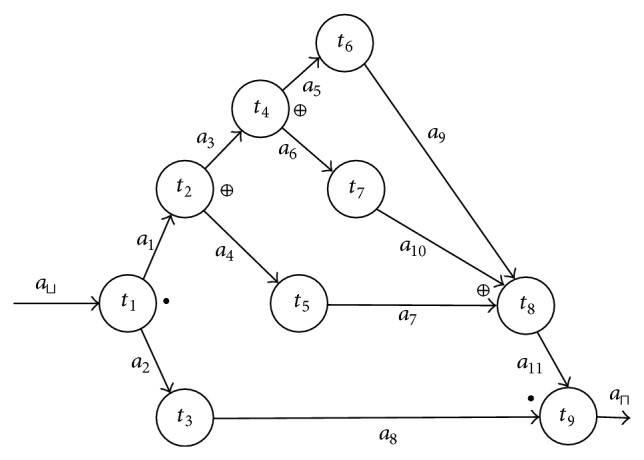
Example of a workflow.

In Figure 1 we present a workflow WG = (*T*, *A*, *A*′, *M*), where *T* = {*t*_1_, *t*_2_,…, *t*_9_},  *A* = {*a*_⊔_, *a*_⊓_, *a*_1_, *a*_2_,…, *a*_11_},  *A*′ = {*a*_⊔_′, *a*_⊓_′, *a*_1_′, *a*_2_′,…, *a*_11_′}, and  *M* = {*t*_1_ : *a*_⊔_′⇝*a*_1_′•*a*_2_′, *t*_2_ : *a*_1_′⇝*a*_3_′ ⊕ *a*_4_′, *t*_3_ : *a*_2_′⇝*a*_8_′, *t*_4_ : *a*_3_′⇝*a*_5_′ ⊕ *a*_6_′, *t*_5_ : *a*_4_′⇝*a*_7_′, *t*_6_ : *a*_5_′⇝*a*_9_′, *t*_7_ : *a*_6_′⇝*a*_10_′, *t*_8_ : *a*_7_′ ⊕ *a*_9_′ ⊕ *a*_10_′⇝*a*_11_′, *t*_9_ : *a*_8_′•*a*_11_′⇝*a*_⊓_′}.

The output logic operator of task *t*_2_  (*t*_2_≺) is XOR (⊕), while the input logic operator of task *t*_9_  (≻*t*_9_) is an AND (•).

The incoming transition for task *t*_2_ is *a*_1_ = (*t*_1_, *t*_2_) and its outgoing transitions are *a*_3_ = (*t*_2_, *t*_4_) and *a*_4_ = (*t*_2_, *t*_5_). Hence the incoming condition for task *t*_2_ is *a*_1_′, while its outgoing condition is *a*_3_′ ⊕ *a*_4_′.

Task *t*_2_ is executed if the EA model *t*_2_ : *a*_1_′⇝*a*_3_′ ⊕ *a*_4_′ is positive, that is, if *a*_1_′ is true and only one of the Boolean terms *a*_3_′, *a*_4_′ is true.

Notice that the workflow from Figure 1 corresponds to the following real situation. Indeed, it can represent the tasks necessary to be executed for a person driving a new car. Let us assume that tasks *t*_*i*_, *i* ∈ {1,…, 9} have the following meanings:

  
*t*_1_: deciding to purchase a new car to own use;
  
*t*_2_: payment of the car;
  
*t*_3_: getting the drivers license;
  
*t*_4_: deciding to pay by credit;
  
*t*_5_: deciding to pay without credit;
  
*t*_6_: getting rental credit;
  
*t*_7_: getting bank credit;
  
*t*_8_: purchasing of the car;
  
*t*_9_: driving the new car.

Clearly, the decision of purchasing a new car to own use implies to pay the car and to get the drivers license. The payment of the car implies the execution of only one of the situations to pay by credit or to pay without credit. And to get credit implies to get a rental credit or a bank credit. It is clear that the purchase of the car depends on the execution of only one of the tasks: decide to pay without credit, get rental credit, and get bank credit.

Hence, the possibility to drive a new car depends on the purchase of the car and in obtaining the drivers license. In other words, the execution of task *t*_9_ depends on the execution of both tasks *t*_3_, *t*_8_.

Many other examples can be given. Indeed, too many situations in our life can be described by workflows. For example, the request for a credit card or a loan application is simple examples of workflows.

**Proposition 5 .**

Let *WG* = (*T*, *A*, *A*′, *M*) be a workflow. Let *a*_*l*_ = (*t*_*i*_, *t*_*j*_) ∈ *A*,  *t*_*i*_, *t*_*j*_ ∈ *T*. If *a*_*l*_′ is true, then *t*_*i*_ is necessarily executed.

**Proof**

Let us assume that *a*_*l*_′ is true. Let *t*_*i*_ : *f*_*E*_*i*__⇝*f*_*C*_*i*__ be the EA model associated to task *t*_*i*_. If task *t*_*i*_ is not executed, then the EA model *t*_*i*_ : *f*_*E*_*i*__⇝*f*_*C*_*i*__ is negative. Since the EA model is negative, all outgoing transitions of task *t*_*i*_ are disabled; in particular *a*_*l*_ is disabled, that is, *a*_*l*_′ is false, wich is a contradiction. Hence task *t*_*i*_ is executed.

**Remark 6 .**

The condition of Proposition 5 is not sufficient. For example in the workflow from Figure 1, if task *t*_2_ is executed, then the EA model *t*_2_ : *a*_1_′⇝*a*_3_′ ⊕ *a*_4_′ is positive. For *a*_1_′ = true, *a*_3_′ = true, *a*_4_′ = false,  and *a*_4_ = (*t*_2_, *t*_5_),  *t*_2_ is executed, but *a*_4_′ is false.

**Remark 7 .**

**Figure 2:**
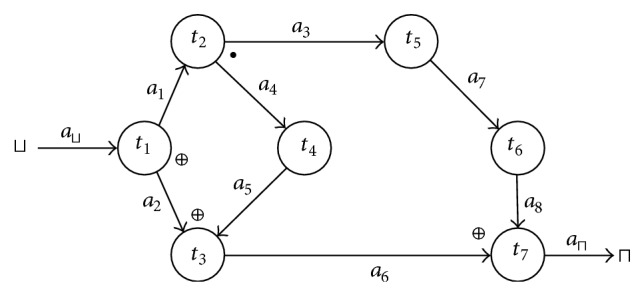
Example of a workflow.

Let us consider the Boolean term *a*_*l*_′ where *a*_*l*_ = (*t*_*i*_, *t*_*j*_) ∈ *A*,  *t*_*i*_, *t*_*j*_ ∈ *T*. If *a*_*l*_′ is true, task *t*_*j*_ is not necessarily executed. For example, in the workflow from Figure 2, let us assume that *a*_⊔_′ = true, *a*_1_′ = true,  *a*_2_′ = false, *a*_3_′ = true, *a*_4_′ = true, *a*_5_′ = true,  *a*_6_′ = true, *a*_7_′ = true, *a*_8_′ = true, and  *a*_⊓_′ = false. Hence, for this assignment the EA model *t*_7_ : *a*_6_′ ⊕ *a*_8_′⇝*a*_⊓_′ is negative, which means that task *t*_7_ is not executed. Nevertheless, *a*_8_ = (*t*_6_, *t*_7_) and *a*_8_′ is true.

Next we introduce the concept of logical termination. This is a very important structural property, since its analysis will allow to verify if a workflow will eventually finish, according to the initial specifications.

**Definition 8 .**

Let WG = (*T*, *A*, *A*′, *M*) be a workflow. One says that WG logically terminates if task *t*_*n*_ is executed whenever task *t*_1_ is executed.

In the following result we establish a necessary and sufficient condition for the logical termination.

**Theorem 9 .**

Let *WG* = (*T*, *A*, *A*′, *M*) be a workflow. Then *WG* logically terminates if and only if *a*_⊓_′ is true whenever *a*_⊔_′ is true.

**Proof**

Let us assume that WG logically terminates; that is, task *t*_*n*_ is executed whenever task *t*_1_ is executed. This means that the EA model *t*_*n*_ : *f*_*E*_*n*__⇝*a*_⊓_′ is positive whenever the EA model *t*_1_ : *a*_⊔_′⇝*f*_*C*_1__ is positive. Bearing in mind that WG starts its execution by executing task *t*_1_, then the EA model *t*_1_ : *a*_⊔_′⇝*f*_*C*_1__ is positive. Hence the EA model *t*_*n*_ : *f*_*E*_*n*__⇝*a*_⊓_′ is also positive. Consequently, *a*_⊔_′,  *f*_*C*_1__,  *f*_*E*_*n*__,  and *a*_⊓_′ are true. Thus, *a*_⊓_′ is true whenever *a*_⊔_′ is true.

Conversely, let us assume that *a*_⊓_′ is true whenever *a*_⊔_′ is true. Let us assume that task *t*_1_ is executed. This means that the EA model *t*_1_ : *a*_⊔_′⇝*f*_*C*_1__ is positive. Bearing in mind that *a*_⊓_′ is true, according to the behavior of the EA models, necessarily *f*_*E*_*n*__ is true. Hence the EA model *t*_*n*_ : *f*_*E*_*n*__⇝*a*_⊓_′ is positive, which means that task *t*_*n*_ is executed. So we can conclude that task *t*_*n*_ is executed whenever task *t*_1_ is executed, which means that WG logically terminates.

**Example 10 .**

It is not hard to check that, in the workflow from Figure 1, *a*_⊓_′ is true whenever *a*_⊔_′ is true. Thus, the workflow logically terminates.

Next we address our study on the dynamism present in a workflow. Obviously the dynamism is associated with the sequential execution of its tasks. In the workflow from Figure 1 the execution of task *t*_1_ implies the execution of both tasks *t*_2_, *t*_3_; the execution of task *t*_2_ implies the execution of only one of the tasks *t*_4_, *t*_5_; the execution of task *t*_4_ implies the execution of only one of the tasks *t*_6_, *t*_7_; the execution of only one of the tasks *t*_5_, *t*_6_, and *t*_7_ implies the execution of task *t*_8_. Finally, the execution of both tasks *t*_3_, *t*_8_ implies the execution of task *t*_9_. Hence, we can state the execution of task *t*_1_ implies the execution of *t*_2_•*t*_3_; the execution of task *t*_2_ implies the execution of *t*_4_ ⊕ *t*_5_; the execution of task *t*_4_ implies the execution of *t*_6_ ⊕ *t*_7_; the execution of *t*_5_ ⊕ *t*_6_ ⊕ *t*_7_ implies the execution of task *t*_8_; the execution of *t*_3_•*t*_8_ implies the execution of task *t*_9_. Notice that when we consider *t*_2_•*t*_3_, the operator • is the output logic operator of task *t*_1_, while when we consider *t*_5_ ⊕ *t*_6_ ⊕ *t*_7_,  ⊕ is the input logic operator of task *t*_8_.

These remarks led us to introduce the following concept.

**Definition 11 .**

Let WG = (*T*, *A*, *A*′, *M*) be a workflow. The compound tasks of WG are the elements of the following form: *t*_*i*_1__ *φt*_*i*_2__ *φ* ⋯ *φt*_*i*_*k*__, *t*_*i*_1__, *t*_*i*_2__,…, *t*_*i*_*k*__ ∈ *T*,  *φ* ∈ {•, ⊗, ⊕}. The set of all compound tasks of WG is denoted by *T*′; that is,

(1) $$\begin{array}{c} T^{\prime }=\left\{t_{i_{1}}\varphi t_{i_{2}}\varphi \cdots \varphi t_{i_{k}}:t_{i_{1}},t_{i_{2}},\ldots ,t_{i_{k}}\in T,\;\varphi \in \left\{•,⊗,⊕\right\}\right\}. \end{array}$$

**Example 12 .**

In the workflow from Figure 1, *T*′ = {*t*_2_•*t*_3_, *t*_4_ ⊕ *t*_5_, *t*_6_ ⊕ *t*_7_, *t*_5_ ⊕ *t*_6_ ⊕ *t*_7_, *t*_3_•*t*_8_}.

**Remark 13 .**

Since every task *t*_*i*_ has associated a Boolean value, according to its execution, it is also natural to attribute a Boolean value to the compound tasks of WG. The natural attribution is the following. Given any compound task of WG,  *t*_*i*_1__ *φt*_*i*_2__ *φ* ⋯ *φt*_*i*_*k*__, *φ* ∈ {•, ⊗, ⊕}.

If *φ* = •, then the Boolean value of *t*_*i*_1__ *φt*_*i*_2__ *φ* ⋯ *φt*_*i*_*k*__ is 1 if and only if the Boolean value of all tasks *t*_*i*_1__, *t*_*i*_2__,…, *t*_*i*_*k*__ is equal to 1.

If *φ* = ⊗, then the Boolean value of *t*_*i*_1__ *φt*_*i*_2__ *φ* ⋯ *φt*_*i*_*k*__ is 1 if and only if there exists at least one of the tasks *t*_*i*_1__, *t*_*i*_2__,…, *t*_*i*_*k*__ whose Boolean value is equal to 1.

If *φ* = ⊕, then the Boolean value of *t*_*i*_1__ *φt*_*i*_2__ *φ* ⋯ *φt*_*i*_*k*__ is 1 if and only if there exists only one of the tasks *t*_*i*_1__, *t*_*i*_2__,…, *t*_*i*_*k*__ with Boolean value equal to 1.

Naturally, we can state that a compound task *t*_*i*_1__ *φt*_*i*_2__ *φ* ⋯ *φt*_*i*_*k*__ is executed if and only if its Boolean value is equal to 1, which means that the compound task *t*_*i*_1__ *φt*_*i*_2__ *φ* ⋯ *φt*_*i*_*k*__ is positive. In other words, *t*_*i*_1__ *φt*_*i*_2__ *φ* ⋯ *φt*_*i*_*k*__ is executed if and only if, one of the following cases holds:

If *φ* = •, all tasks *t*_*i*_1__, *t*_*i*_2__,…, *t*_*i*_*k*__ are executed.

If *φ* = ⊗, at least one of the tasks *t*_*i*_1__, *t*_*i*_2__,…, *t*_*i*_*k*__ is executed.

If *φ* = ⊕, only one of the tasks *t*_*i*_1__, *t*_*i*_2__,…, *t*_*i*_*k*__ is executed.

In what follows, we introduce a new type of task models that we designate by compound task models.

**Definition 14 .**

Let WG = (*T*, *A*, *A*′, *M*) be a workflow. Let *t*_*i*_, *t*_*j*_, *t*_*i*_1__, *t*_*i*_2__,…, *t*_*i*_*k*__, *t*_*j*_1__, *t*_*j*_2__,…, *t*_*j*_*l*__ ∈ *T*, *φ*, *ψ*{•, ⊗, ⊕}. A compound task model is an implication with one of the following forms:

1. *t*_*i*_↪*t*_*j*_1__ *ψt*_*j*_2__ *ψ* ⋯ *ψt*_*j*_*l*__;
2. *t*_*i*_1__ *φt*_*i*_2__ *φ* ⋯ *φt*_*i*_*k*__↪*t*_*j*_;
3. *t*_*i*_1__ *φt*_*i*_2__ *φ* ⋯ *φt*_*i*_*k*__↪*t*_*j*_1__ *ψt*_*j*_2__ *ψ* ⋯ *ψt*_*j*_*l*__.

Usually we represent a compound task model by *t*_*I*_*i*__↪*t*_*O*_*i*__, where *t*_*I*_*i*__ is called the incoming task and *t*_*O*_*i*__ is called the outgoing task. We say that a compound task model *t*_*I*_*i*__↪*t*_*O*_*i*__ is positive if both incoming and outgoing tasks are positive, that is, if both tasks *t*_*I*_*i*__, *t*_*O*_*i*__ are executed.

In particular, the implication of the form *t*_*i*_↪*t*_*j*_ is called a simple task model. Clearly, it is positive if both tasks *t*_*i*_, *t*_*j*_ are executed.

The set of all simple and compound task models present in WG is called the set of task models of WG and is denoted by TM.

The task models have the behavior with two distinct modes: if its incoming task is true, necessarily its outgoing task is true; if the incoming task is false, the outgoing task is false. In other words, if *t*_*I*_*i*__↪*t*_*O*_*i*__ is a compound task model, then *t*_*I*_*i*__ is executed if and only if *t*_*O*_*i*__ is executed.

Notice that, in a compound task model *t*_*I*_*i*__↪*t*_*O*_*i*__, at least one of the tasks *t*_*I*_*i*__,  *t*_*O*_*i*__ is compound.

**Example 15 .**

In the workflow from Figure 1, the set of its task models is TM = {*t*_1_↪*t*_2_•*t*_3_, *t*_2_↪*t*_4_ ⊕ *t*_5_, *t*_4_↪*t*_6_ ⊕ *t*_7_, *t*_5_ ⊕ *t*_6_ ⊕ *t*_7_↪*t*_8_, *t*_3_•*t*_8_↪*t*_9_}.

From now on, we use the symbol ↔ with the following meaning: *X*↔*Y* means that the compound statements *X* and *Y* are logically equivalent.

According to simple rules of Logic and taking into account the behavior of the task models, we can infer the following result. And the establishment of this result allows us to identify new task models present in the workflow.

In what follows we establish some properties that will allow us to create new task models based on the existing ones.

**Proposition 16 .**

Let *WG* = (*T*, *A*, *A*′, *M*) be a workflow. Suppose that the task models *t*_*I*_*i*__↪*t*_*O*_*i*__ and *t*_*O*_*i*__↪*t*_*O*_*j*__ belong to *TM*. Then the model *t*_*I*_*i*__↪*t*_*O*_*j*__ still hods in *WG*.

**Proof**

The proof is trivial.

**Theorem 17 .**

Let *WG* = (*T*, *A*, *A*′, *M*) be a workflow.

(a) If both task models
  (2) $$\begin{array}{c} t_{I_{i}}↪t_{O_{i}}, \end{array}$$
  (3) $$\begin{array}{c} t_{I_{j}}↪t_{O_{j}} \end{array}$$
  belong to *TM*, where *t*_*O*_*i*__↔*t*_*I*_*j*__, then the model *t*_*I*_*i*__↪*t*_*O*_*j*__ still holds in *WG*.
(b) If both task models *t*_*I*_*i*__↪*t*_*O*_*i*__ and *t*_*I*_*j*__↪*t*_*O*_*j*__ belong to *TM*, where *t*_*O*_*i*__↔*t*_*L*_ *φt*_*I*_*j*__,  *φ* ∈ {•, ⊗, ⊕}, then the compound task model *t*_*I*_*i*__↪*t*_*L*_ *φt*_*O*_*j*__ still holds in *WG*.
(c) If both task models *t*_*I*_*i*__↪*t*_*O*_*i*__ and *t*_*O*_*j*__↪*t*_*I*_*j*__ belong to *TM*, where *t*_*O*_*i*__↔*t*_*L*_ *φt*_*I*_*j*__,  *φ* ∈ {•, ⊗, ⊕}, then the compound task model *t*_*I*_*i*__↪*t*_*L*_ *φt*_*O*_*j*__ still holds in *WG*.
(d) If both task models *t*_*I*_*i*__↪*t*_*O*_*i*__ and *t*_*I*_*j*__↪*t*_*O*_*j*__ belong to *TM*, where *t*_*I*_*i*__↔*t*_*L*_ *φt*_*I*_*j*__,  *φ* ∈ {•, ⊗, ⊕}, then the compound task model *t*_*L*_ *φt*_*O*_*j*__↪*t*_*O*_*i*__ still holds in *WG*.
(e) If both task models *t*_*I*_*i*__↪*t*_*O*_*i*__ and *t*_*O*_*j*__↪*t*_*I*_*j*__ belong to *TM*, where *t*_*I*_*i*__↔*t*_*L*_ *φt*_*I*_*j*__,  *φ* ∈ {•, ⊗, ⊕}, then the compound task model *t*_*L*_ *φt*_*O*_*j*__↪*t*_*O*_*i*__ still holds in *WG*.

**Proof**

(a) Let us assume that both task models (2) and (3) belong to TM. Notice that if either (2) or (3) is negative, since *t*_*O*_*i*__↔*t*_*I*_*j*__, then necessarily both task models (2) and (3) are negative. So, we just need to verify the result when both task models (2) and (3) are positive.

Let us assume that both tasks models (2) and (3) are positive. Since (2) is positive, necessarily both *t*_*I*_*i*__, *t*_*O*_*i*__ are true. In other words, some of the tasks from *t*_*I*_*i*__, *t*_*O*_*i*__ are executed allowing that *t*_*I*_*i*__, *t*_*O*_*i*__ are executed. Bearing in mind that *t*_*O*_*i*__↔*t*_*I*_*j*__, then *t*_*I*_*j*__ is true. So according to the behavior of the task models, necessarily *t*_*O*_*j*__ is true. Hence we can state that *t*_*O*_*j*__ is executed, whenever *t*_*I*_*j*__ is executed. Therefore the model *t*_*I*_*i*__↪*t*_*O*_*j*__ still holds in WG.

Now, in order to prove (b) and (c), we start with the following argument. Bearing in mind that *t*_*O*_*i*__↔*t*_*L*_ *φt*_*I*_*j*__, according to the behavior of the task models, we can consider that *t*_*O*_*i*__↪*t*_*L*_ *φt*_*I*_*j*__ still holds in WG. Since *t*_*I*_*i*__↪*t*_*O*_*i*__ and *t*_*O*_*i*__↪*t*_*L*_ *φt*_*I*_*j*__ holds in WG, according to Proposition 16 we can conclude that

(4) $$\begin{array}{c} t_{I_{i}}↪t_{L}\varphi t_{I_{j}} \end{array}$$

still holds in WG.

(b) Taking into account that *t*_*I*_*j*__↪*t*_*O*_*j*__ belong to TM, and consequently *t*_*I*_*j*__,  *t*_*O*_*j*__ have the same Boolean value, we can replace *t*_*I*_*j*__ by *t*_*O*_*j*__ in (4), obtaining the new model *t*_*I*_*i*__↪*t*_*L*_ *φt*_*O*_*j*__, which means that *t*_*I*_*i*__↪*t*_*L*_ *φt*_*O*_*j*__ still holds in WG.

(c) Taking into account that *t*_*O*_*j*__↪*t*_*I*_*j*__ belong to TM, and consequently *t*_*O*_*j*__, *t*_*I*_*j*__ have the same Boolean value, we can replace *t*_*I*_*j*__ by *t*_*O*_*j*__ in (4), obtaining the new model *t*_*I*_*i*__↪*t*_*L*_ *φt*_*O*_*j*__, which means that *t*_*I*_*i*__↪*t*_*L*_ *φt*_*O*_*j*__ still holds in WG.

Analogously, in order to prove (d) and (e) we start by making the next statement. Taking into account that *t*_*I*_*i*__↔*t*_*L*_ *φt*_*I*_*j*__, according to the behavior of the compound task models, we can consider that *t*_*L*_ *φt*_*I*_*j*__↪*t*_*I*_*i*__ holds in WG. Since *t*_*L*_ *φt*_*I*_*j*__↪*t*_*I*_*i*__ and *t*_*I*_*i*__↪*t*_*O*_*i*__ hold in WG, according to Proposition 16 we can conclude that

(5) $$\begin{array}{c} t_{L}\varphi t_{I_{j}}↪t_{O_{i}} \end{array}$$

still holds in WG.

(d) Bearing in mind that *t*_*I*_*j*__↪*t*_*O*_*j*__ holds in WG, and consequently *t*_*I*_*j*__, *t*_*O*_*j*__ have the same Boolean value, we can replace *t*_*I*_*j*__ by *t*_*O*_*j*__ in (5), obtaining the new model *t*_*L*_ *φt*_*O*_*j*__↪*t*_*O*_*i*__, which means that *t*_*L*_ *φt*_*O*_*j*__↪*t*_*O*_*i*__ still holds in WG.

(e) Bearing in mind that *t*_*O*_*j*__↪*t*_*I*_*j*__ holds in WG, and consequently *t*_*O*_*j*__, *t*_*I*_*j*__ have the same Boolean value, we can replace *t*_*I*_*j*__ by *t*_*O*_*j*__ in (5), obtaining the new model *t*_*L*_ *φt*_*O*_*j*__↪*t*_*O*_*i*__, which means that *t*_*L*_ *φt*_*O*_*j*__↪*t*_*O*_*i*__ still holds in WG.

The previous results allow us to identify new task models based on the existing ones, as it is described below.

**Definition 18 .**

Let WG = (*T*, *A*, *A*′, *M*) be a workflow. An extended task model is a model obtained by applying a finite sequence of some of the properties established in Proposition 16 and Theorem 17. One adopts the notation TM′ to represent the set of all extended task models of WG.

**Example 19 .**

In the workflow from Figure 1, bearing in mind that *t*_1_↪*t*_2_•*t*_3_, *t*_2_↪*t*_4_ ⊕ *t*_5_ ∈ TM, according to Theorem 17 we can conclude that the model *t*_1_↪(*t*_4_ ⊕ *t*_5_)•*t*_3_ still holds in WG. Therefore, we can state that *t*_1_↪(*t*_4_ ⊕ *t*_5_)•*t*_3_ is an extended task model of WG.

Notice we adopt the same notation of the task models to represent the extended task models. Furthermore, the extended task models verify the same properties of the task models. In particular, given an extended task model *B*↪*C*, necessarily both *B*, *C* have the same Boolean value.

Naturally when we consider the set of all task models and extended task models presented in WG, we obtain all possible models that can be generated by the task models of the workflow. This set of task models will be designated by the closure of TM. In certain sense, we can state that the set of all task models from WG is a set of generators of all possible models of WG.

**Definition 20 .**

Let WG = (*T*, *A*, *A*′, *M*) be a workflow. One defines the closure of TM as the set of all task models and extended task models in WG. This set is denoted by TM^*^. In other words, TM^*^ = TM ∪ TM′.

**Example 21 .**

As we saw in Example 15 in the workflow from Figure 1, TM = {*t*_1_↪*t*_2_•*t*_3_, *t*_2_↪*t*_4_ ⊕ *t*_5_, *t*_4_↪*t*_6_ ⊕ *t*_7_, *t*_5_ ⊕ *t*_6_ ⊕ *t*_7_↪*t*_8_, *t*_3_•*t*_8_↪*t*_9_}. Since *t*_1_↪*t*_2_•*t*_3_, *t*_2_↪*t*_4_ ⊕ *t*_5_ ∈ TM, according to Theorem 17 we can deduce that *t*_1_↪(*t*_4_ ⊕ *t*_5_)•*t*_3_ ∈ TM^*^. Now bearing in mind that *t*_4_↪*t*_6_ ⊕ *t*_7_ ∈ TM, applying again Theorem 17 we can conclude that *t*_1_↪((*t*_6_ ⊕ *t*_7_) ⊕ *t*_5_)•*t*_3_ ∈ TM^*^. As (*t*_6_ ⊕ *t*_7_) ⊕ *t*_5_↔*t*_5_ ⊕ *t*_6_ ⊕ *t*_7_ we can state that *t*_1_↪(*t*_5_ ⊕ *t*_6_ ⊕ *t*_7_)•*t*_3_ ∈ TM^*^. Bearing in mind that *t*_5_ ⊕ *t*_6_ ⊕ *t*_7_↪*t*_8_, applying once more Theorem 17 we infer that *t*_1_↪*t*_8_•*t*_3_ ∈ TM^*^. As *t*_8_•*t*_3_↔*t*_3_•*t*_8_, applying again Theorem 17 we conclude that *t*_1_↪*t*_9_ ∈ TM^*^.

Notice the workflow from Figure 1 logically terminates and *t*_1_↪*t*_9_ ∈ TM^*^. Furthermore, we studied many other examples of workflows that logically terminates and simultaneously *t*_1_↪*t*_*n*_ ∈ TM^*^. The analysis of these different cases led us to formulate the following conjecture.

**Conjecture 22 (see [21]).**

Given a workflow *WG* = (*T*, *A*, *A*′, *M*), then *WG* logically terminates if and only if *t*_1_↪*t*_*n*_ ∈ *TM* ^*^.

After the analysis of many cases, we start believing that the conjecture was true. Nevertheless it is false, as the following example proves. Let us consider the following workflow.

**Example 23 .**

**Figure 3:**
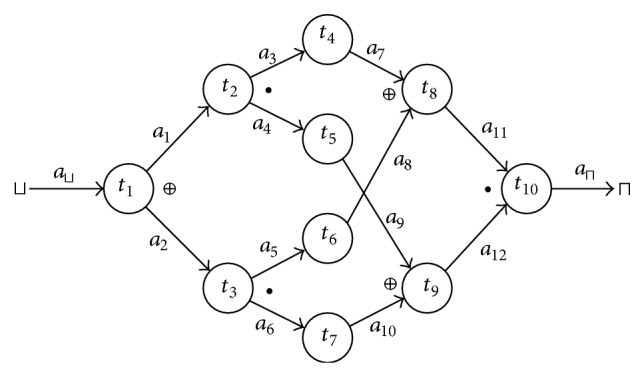
Example of a workflow.

It is not hard to check that the workflow from Figure 3 logically terminates; nevertheless the condition *t*_1_↪*t*_*n*_ ∈ TM^*^ is not valid.

Indeed the condition of the Conjecture 22 is not necessary. However it is sufficient, as we prove in the following result.

**Theorem 24 .**

Let *WG* = (*T*, *A*, *A*′, *M*) be a workflow. If *t*_1_↪*t*_*n*_ ∈ *TM* ^*^, then *WG* logically terminates.

**Proof**

Let us assume that *t*_1_↪*t*_*n*_ ∈ TM^*^.

*Case 1*. Suppose that *t*_1_↪*t*_*n*_ ∈ TM. This means that WG has the following structure:

(6) $$\begin{array}{c} ⊔\overset{a_{⊔}}{⟶}t_{1}\overset{a_{⊓}}{⟶}t_{⊓}. \end{array}$$

In this case, *t*_1_ = *t*_*n*_. Since WG starts its execution by executing task *t*_1_ we can conclude that task *t*_*n*_ is executed whenever task *t*_1_ is executed, which means that WG logically terminates.

*Case 2*. Suppose that *t*_1_↪*t*_*n*_ ∈ TM^*^∖TM = TM′. So, *t*_1_↪*t*_*n*_ was obtained by applying some of the results established in Proposition 16 and Theorem 17. Since *t*_1_↪*t*_*n*_ ∈ TM^*^ and the workflow starts its execution by executing task *t*_1_, that is, by asserting *t*_1_ to be true, according to the behavior of the extended models, necessarily *t*_*n*_ is still asserted to be true. This means that task *t*_*n*_ is executed. Hence, task *t*_*n*_ is executed whenever task *t*_1_ is executed. Thus WG logically terminates.

## 3. Conclusions

In this paper we develop a formalism to describe and analyse the structure of workflows, based on graphs and Propositional Logic. Indeed, we describe the structure of a workflow as a graph whose vertices represent tasks and the arcs are associated to workflow transitions. To each task an input/output logic operator is associated and this logic operator can be the logical AND (•), the OR (⊗), or the XOR—exclusive-or—(⊕). Furthermore, we associate a Boolean term to each transition present in the workflow.

It is important to point out that our main emphasis is the analysis of a workflow through the study of its task models which allows us to describe the dynamism of the workflow in a very simple and intuitive way.

Another relevant aspect of our approach is the introduction of the concept of compound tasks. This concept allows us to identify new task models based on the existing ones. Through these new task models we are able to describe the dynamism present in a workflow in a very simple way. Clearly, the study of the dynamism of a workflow is equivalent to analyse the sequential execution of its tasks.

We still analyze the concept of logical termination and we provide necessary and sufficient conditions under which this property is valid.

Finally, given a workflow WG we provide a counter-example which shows that the conjecture of *t*_1_↪*t*_*n*_ ∈ TM^*^ being a necessary and sufficient condition under which WG logically terminates is false. In fact, the condition is necessary; nevertheless it is not sufficient.
